# Supplementary material for: The antibiotic resistance reservoir of the lung microbiome expands with age in a population of critically ill patients
Source: Nat Commun. 2024 Jan 2;15:92. doi: 10.1038/s41467-023-44353-1 (PMC10762195; doi:10.1038/s41467-023-44353-1)
Supplement: Supplementary file 1 — Supplementary Information [file 41467_2023_44353_MOESM1_ESM.pdf]

Supplementary Materials

The antibiotic resistance reservoir of the lung microbiome expands with age in a population of critically ill patients

Supplemental Table 1. Demographics and clinical characteristics of the patients included in the analysis.

	Children n=261	Adults n=88	Total n=349	p-value*
Age (years), median (range)	1 (0-17)	63 (21-94)	3 (0-94)	---
0-2 years	170 (65%)	---	170 (65%)	---
3-10 years	51 (20%)	---	51 (20%)	---
11-18 years	40 (15%)	---	40 (15%)	---
19-39 years	---	11 (13%)	11 (13%)	---
40-49 years	---	6 (7%)	6 (7%)	---
50-59 years	---	20 (23%)	20 (23%)	---
60-69 years	---	24 (27%)	24 (27%)	---
70-79 years	---	15 (17%)	15 (17%)	---
≥80 years	---	12 (14%)	12 (14%)	---
Sex, no. (%)				
Female	112 (43%)	30 (34%)	142 (41%)	0.18
Male	149 (57%)	58 (66%)	206 (59%)	
Race/Ethnicity, no. (%)				3.0×10^{-8}
Non-Hispanic White	132 (51%)	45 (51%)	177 (51%)	
Hispanic White	38 (15%)	8 (9%)	46 (13%)	
Asian	14 (5%)	26 (30%)	40 (12%)	
Black or African American	49 (19%)	5 (6%)	54 (16%)	
Other or Multiracial	15 (6%)	4 (5%)	19 (6%)	
Missing	15 (6%)	0 (0%)	15 (4%)	
LRTI Status, no. (%)				2.9×10^{-6}

CA-LRTI	159 (61%)	35 (40%)	194 (56%)	
HA-LRTI	15 (6%)	22 (25%)	37 (11%)	
Indeterminate	37 (14%)	14 (16%)	51 (15%)	
No Evidence	50 (19%)	17 (19%)	67 (19%)	
Received antibiotics on or prior to sample collection	235 (90%)	79 (90%)	314 (90%)	1.00
Days from intubation to specimen collection				---
≤1 day	261 (100%)	60 (68%)	321 (92%)	
2 days	---	20 (23%)	20 (6%)	
3 days	---	8 (9%)	8 (2%)	
Geographic Location, no. (%)				---
Midwest (2 sites)	111 (43%)	---	111 (32%)	
West (3 sites)	76 (29%)	88 (100%)	164 (47%)	
Northeast (2 site)	48 (18%)	---	48 (14%)	
South (1 site)	26 (10%)	---	26 (7%)	

*p-value obtained by two-sided Pearson's Chi-square test.

Abbreviations: LRTI, lower respiratory tract infection; CA-LRTI, community-acquired LRTI; HA-LRTI, hospital-acquired LRTI.

Supplemental Table 2. Patients with an antimicrobial resistance gene (ARG) for each ARG class, stratified by children and adults.

	**Patients with ARGs**		
ARG class	**Children**<br>n=261<br>n (%, 95% CI)	**Adults**<br>n=88<br>n (% [95% CI])	**p-value***
Aminoglycoside	5 (2%, 0.6-4%)	18 (20%, 13-30%)	6.2×10^{-9}
Beta-lactam	35 (13%, 10-18%)	27 (31%, 21-41%)	4.6×10^{-4}
Chloramphenicol	5 (2%, 0.6-4%)	8 (9%, 4-17%)	6.0×10^{-3}
Fluoroquinolone	5 (2%, 0.6-4%)	7 (8%, 3-16%)	0.019
Fosfomycin	0 (0%, 0-1.4%)	1 (1%, 0.03-6%)	0.25
MLS	18 (7%, 4-11%)	12 (14%, 7-23%)	0.084
Tetracycline	7 (3%, 1-5%)	12 (14%, 7-23%)	2.7×10^{-4}
TMP-SMX	1 (0%, 0.01-2%)	1 (1%, 0.03-6%)	0.44

*p-value obtained by two-sided Pearson's Chi-square test and Fisher's exact test for comparisons with counts <5.

Abbreviations: ARG, antimicrobial resistance genes; 95% CI, 95% confidence interval; MLS, macrolide-lincosamide-streptogramin; TMP-SMX, trimethoprim-sulfamethoxazole.

Supplemental Table 3. Multivariable logistic regression model evaluating the association of continuous age with the presence of ARGs while accounting for sex, race/ethnicity, and lower respiratory tract infection (LRTI) status (n = 349 patients).

	**uOR (95% CI)**	**aOR (95% CI)**	**p-value***
Continuous age (years)	1.02 (1.01-1.03)	1.02 (1.01-1.03)	0.004
Sex (female vs male)	0.94 (0.58-1.53)	0.99 (0.59-1.68)	0.98
Race/Ethnicity (ref: non-Hispanic White)			
Hispanic White	1.29 (0.63-2.64)	1.59 (0.74-3.42)	0.24
Black or African American	0.88 (0.43-1.78)	1.21 (0.58-2.55)	0.61
Asian	1.66 (0.81-3.42)	1.08 (0.47-2.48)	0.86
Other	0.52 (0.14-1.86)	0.55 (0.15-2.07)	0.38
LRTI Status (ref: no LRTI)			
Indeterminate	1.30 (0.58-2.93)	1.30 (0.56-3.03)	0.55
CA-LRTI	0.77 (0.4-1.48)	0.82 (0.41-1.63)	0.56
HA-LRTI	3.09 (1.31-7.29)	2.24 (0.89-5.62)	0.09
Intubation to specimen collection (ref: ≤1 day)			
2 days	2.97 (1.19-7.42)	0.97 (0.32-2.92)	0.96
3 days	4.96 (1.16-21.23)	2.60 (0.55-12.17)	0.23

*p-value obtained by two-sided Wald test.

Abbreviations: uOR, unadjusted odds ratio; aOR, adjusted odds ratio; CI, confidence interval; ref, referent; CA-LRTI, community-acquired LRTI; HA-LRTI, hospital-acquired LRTI.

Supplemental Table 4. Multivariable logistic regression model in the pediatric cohort only, evaluating the association of age with the presence of ARGs, accounting for the region of the enrollment site, as defined by the U.S. Census Bureau (n = 349 patients).

	uOR (95% CI)	aOR (95% CI)	p-value*
Age (ref: 0-2 years)			
3-10 years	0.34 (0.11-1.01)	0.26 (0.08-0.82)	0.021
11-18 years	2.24 (1.05-4.76)	2.06 (0.92-4.60)	0.079
Presence of a complex chronic condition	0.89 (0.48-1.64)	0.8 (0.41-1.59)	0.53
Enrollment site region (ref: Midwest)			
South	2.26 (0.77-6.66)	2.39 (0.79-7.18)	0.12
West	2.56 (1.17-5.57)	3.09 (1.38-6.93)	0.006
Northeast	3.10 (1.33-7.26)	3.84 (1.56-9.47)	0.004

*p-value obtained by two-sided Wald test.

Abbreviations: uOR, unadjusted odds ratio; aOR, adjusted odds ratio; CI, confidence interval; ref, referent.

Supplemental Table 5. Culture and antimicrobial susceptibility testing results for children and adults (n = 349 patients). Susceptibility testing for (A) gram-positive pathogens and (B) gram negative pathogens are shown. All specimen sources are from tracheal aspirates, unless otherwise indicated. Abbreviations: TMP-SMX, trimethoprim-sulfamethoxazole; S, susceptible; SDD, susceptible-dose dependent; I, intermediate susceptibility; R, resistant.

A.

Patient ID	Gram-positive Pathogen	Amoxicillin / Ampicillin / Penicillin	Nafcillin / Oxacillin	Clindamycin	Erythromycin	Ceftriaxone / Ceftazidime	TMP-SMX	Vancomycin
**Children**								
1028330	*Streptococcus, beta hemolytic*	No susceptibilities available						
1028513	*Streptococcus, beta hemolytic*	No susceptibilities available						
1030846	*Streptococcus pneumoniae* (pleural fluid)	S		S		S	S	S
1036430	*Staphylococcus aureus (MRSA)*		R	R	R		R	S
1037041	*Streptococcus pneumoniae* (pleural fluid)	S		R	R	S	S	S
1038522	*Streptococcus, viridans*	No susceptibilities available						
1040253	*Staphylococcus aureus (MRSA)*		R	S	S		S	S
1041664	*Streptococcus pyogenes*	No susceptibilities available						
1044367	*Streptococcus pneumoniae*	S		S	R	S		
1046056	*Streptococcus pyogenes*	No susceptibilities available						
1046750	*Staphylococcus aureus (MRSA)* (pleural fluid)	R	R	S	S		S	S
1047433	*Streptococcus, viridans*	No susceptibilities available						

Patient ID	Gram-positive Pathogen	Amoxicillin / Ampicillin / Penicillin	Nafcillin / Oxacillin	Clindamycin	Erythromycin	Ceftriaxone / Ceftazidime	TMP-SMX	Vancomycin
**Adults**								
212	*Staphylococcus aureus*	R	S	R	R		S	S
225	*Staphylococcus aureus*	R	R	R	R		S	S
252	*Staphylococcus aureus*			R	R		S	S
252	*Streptococcus agalactiae*	No susceptibilities available						
252	*Streptococcus viridans*	No susceptibilities available						
288	*Staphylococcus aureus*	R	R	S	R		S	S
289	*Staphylococcus aureus*	R	S	S	S		S	S
304	*Staphylococcus aureus*	R	S	S	S		S	S
314	*Staphylococcus aureus*	R	S	S	S		S	
315	*Streptococcus pneumoniae*	I		S	S	S	S	S
334	*Streptococcus pneumoniae*	S			S			
337	*Streptococcus pneumoniae*	R		S	S	I	R	S

B.

Patient ID	Gram-negative Pathogen	Ampicillin	Ampicillin-Sulbactam	Cefazolin	Ceftriaxone / Ceftazidime	Ertapenem	Gentamicin / Amikacin	Piperacillin-Tazobactam	TMP-SMX	Meropenem
**Children**										
1027570	*Haemophilus influenzae*	S								
1028607	*Haemophilus influenzae*	S								
1028901	*Haemophilus influenzae*	R								
1028941	*Moraxella catarrhalis*				S				S	
1028941	*Haemophilus influenzae*	S								
1030846	*Moraxella catarrhalis*				S				S	
1036430	*Moraxella catarrhalis*	R								
1036430	*Haemophilus influenzae*	S								
1037426	*Moraxella catarrhalis*	R								
1037426	*Haemophilus influenzae*	S								
1037465	*Haemophilus influenzae*	R								
1038522	*Moraxella catarrhalis*	R								
1038766	*Haemophilus influenzae*	R								
1039141	*Moraxella catarrhalis*	R								
1039815	*Haemophilus influenzae*	S								
1040123	*Moraxella catarrhalis*	No susceptibilities available								
1040202	*Moraxella catarrhalis*	R								
1040644	*Moraxella catarrhalis*	R								
1040644	*Haemophilus influenzae*	S								
1044367	*Stenotrophomonas maltophilia*				S				S	
1044367	*Klebsiella pneumonia*	R	S	S	S	S	S	S	S	S
1047433	*Haemophilus influenzae*	S								
1047433	*Moraxella catarrhalis*	R								
1047479	*Haemophilus influenzae*	R								
1047591	*Moraxella catarrhalis*	No susceptibilities available								
1047709	*Haemophilus influenzae*	R								
1048263	*Moraxella catarrhalis*	R								

Patient ID	Gram-negative Pathogen	Ampicillin	Ampicillin-Sulbactam	Cefazolin	Ceftriaxone / Ceftazidime	Ertapenem	Gentamicin / Amikacin	Piperacillin-Tazobactam	TMP-SMX	Meropenem
**Adults**										
213	*Escherichia coli*		S	S	S		S	S	S	
213	*Klebsiella pneumoniae*		S		S		S	S	S	
232	*Klebsiella pneumoniae*		I	I	S		S	SDD	S	
251	*Klebsiella pneumoniae*		S	S	S		S	S	S	
252	*Enterobacter cloacae*			R		S	S		S	
257	*Enterobacter aerogenes*			S		S	S		S	
257	*Enterobacter cloacae*					S	S		S	
268	*Pseudomonas aeruginosa*						R	S		S
268	*Stenotrophomonas maltophila*				R				S	
278	*Moraxella catarrhalis*									
290	*Moraxella catarrhalis*									
297	*Pseudomonas aeruginosa*							S		
297	*Morganella morganii*		S		S		S	S	S	
297	*Klebsiella spp.*	No susceptibilities available								
297	*Enterobacter aerogenes*				S	S	S		S	
298	*Enterobacter cloacae*			R		S	S		S	
343	*Burkholderia cepaciae*									S
350	*Serratia marcescans*		R	R	S		S	S	S	

Supplemental Table 6. Multivariable logistic regression model evaluating the association of binary age (adult vs children) with the presence of ARGs, accounting for differences in the bacterial microbiome. The model includes the logarithmic total bacterial abundance [log (NT rpm +0.01)] per patient sample, bacterial alpha diversity (measured by Shannon diversity index), and lower respiratory tract infection (LRTI) status (n = 349 patients). Each model also included one of the bacterial genera detected by metagenomics next-generation sequencing that was found to be differentially abundant between patients with ARGs and patients without ARGs (*Bacteroides spp*., *Fusobacterium spp*., *Prevotella spp*., *Staphylococcus spp*., *Enterococcus spp.*, *Neisseria spp.*, and *Pseudomonas spp.*). Age and total bacterial abundance remained significant with a two-sided Wald test p-values (<0.05) across all models. Source data, including the exact p-values, are provided as a Source Data file.

	***Bacteroides* Model**	***Enterococcus* Model**	***Fusobacterium* Model**
	**aOR (95% CI)**	**aOR (95% CI)**	**aOR (95% CI)**
Age (adults vs children)	2.00 (1.95-5.48)	1.62 (0.82-3.21)	2.22 (1.17-4.21)
Bacterial abundance	1.15 (1.06-1.24)	1.16 (1.08-1.25)	1.14 (1.06-1.23)
Alpha diversity	0.87 (0.65-1.16)	0.88 (0.66-1.17)	0.84 (0.63-1.13)
LRTI Status (ref: no LRTI)			
Indeterminate	1.07 (0.45-2.56)	1.24 (0.52-3.00)	1.17 (0.49-2.81)
CA-LRTI	0.73 (0.35-1.52)	0.78 (0.38-1.62)	0.75 (0.36-1.56)
HA-LRTI	2.00 (0.77-5.19)	2.09 (0.80-5.48)	2.23 (0.85-5.81)
Bacterial genera			
Bacteroides spp.	1.89 (0.96-3.69)	---	---
Enterococcus spp.	---	2.23 (1.08-4.59)	---
Fusobacterium spp.	---	---	2.18 (1.13-4.22)

	Neisseria Model	*Prevotella* Model	*Pseudomonas* Model
	**aOR (95% CI)**	**aOR (95% CI)**	**aOR (95% CI)**
Age (adults vs children)	2.05 (1.08-3.89)	2.13 (1.13-4.03)	1.93 (0.99-3.77)
Bacterial abundance	1.18 (1.09-1.28)	1.15 (1.06-1.24)	1.18 (1.09-1.27)
Alpha diversity	1.00 (0.75-1.33)	0.87 (0.64-1.17)	0.93 (0.70-1.23)
LRTI Status (ref: no LRTI)			
Indeterminate	1.14 (0.48-2.70)	1.15 (0.48-2.73)	1.13 (0.48-2.69)
CA-LRTI	0.73 (0.35-1.51)	0.76 (0.37-1.56)	0.77 (0.37-1.59)
HA-LRTI	1.86 (0.72-4.76)	2.07 (0.79-5.39)	1.87 (0.72-4.82)
Bacterial genera			
Neisseria spp.	0.81 (0.45-1.48)	---	---
Prevotella spp.	---	1.66 (0.87-3.16)	---
Pseudomonas spp.	---	---	1.34 (0.69-2.59)

	***Staphylococcus* Model**
	**aOR (95% CI)**
Age (adults vs children)	1.67 (0.84-3.30)
Bacterial abundance	1.18 (1.09-1.27)
Alpha diversity	0.91 (0.69-1.20)
LRTI Status (ref: no LRTI)	
Indeterminate	1.18 (0.49-2.82)
CA-LRTI	0.75 (0.36-1.55)
HA-LRTI	2.01 (0.78-5.20)
Bacterial genera	
Staphylococcus spp.	1.86 (0.99-3.51)

Abbreviations: aOR, adjusted odds ratio; CI, confidence interval; NT rpm, nucleotide reads per million.

Supplemental Table 7. Sensitivity analyses with multivariable logistic regression model evaluating the association of continuous age (years) with the presence of ARGs, accounting for differences in the bacterial microbiome. The model includes the logarithmic total bacterial abundance [log (NT rpm +0.01)] per patient sample, bacterial alpha diversity (measured by Shannon diversity index), and lower respiratory tract infection (LRTI) status (n = 349 patients). Additional models also included one of the bacterial genera detected by metagenomics next-generation sequencing that was found to be differentially abundant between patients with ARGs and patients without ARGs (*Bacteroides spp*., *Enterococcus spp.*, *Fusobacterium spp*., *Neisseria spp.*, *Prevotella spp*., *Pseudomonas spp.*, and *Staphylococcus spp.*). Age and total bacterial abundance remained significant with a two-sided Wald test p-values (<0.05) across all models. Source data, including the exact p-values, are provided as a Source Data file.

	**Primary Model**	***Bacteroides* Model**	***Enterococcus* Model**
	**aOR (95% CI)**	**aOR (95% CI)**	**aOR (95% CI)**
Age (years)	1.02 (1.00-1.03)	1.01 (1.00-1.02)	1.01 (1.00-1.02)
Bacterial abundance	1.17 (1.09-1.26)	1.15 (1.06-1.24)	1.16 (1.08-1.25)
Alpha diversity	0.94 (0.72-1.22)	0.86 (0.64-1.15)	0.87 (0.65-1.15)
LRTI Status (ref: no LRTI)			
Indeterminate	1.18 (0.50-2.81)	1.13 (0.47-2.69)	1.27 (0.53-3.05)
CA-LRTI	0.78 (0.38-1.60)	0.76 (0.37-1.58)	0.80 (0.38-1.66)
HA-LRTI	1.79 (0.69-4.62)	1.92 (0.74-5.01)	1.96 (0.75-5.16)
Bacterial genera			
Bacteroides spp.	---	1.78 (0.91-3.52)	---
Enterococcus spp.	---	---	2.04 (0.98-4.23)

	***Fusobacterium* Model**	***Neisseria* Model**	***Prevotella* Model**
	**aOR (95% CI)**	**aOR (95% CI)**	**aOR (95% CI)**
Age (years)	1.02 (1.01-1.03)	1.01 (1.00-1.03)	1.02 (1.00-1.03)
Bacterial abundance	1.14 (1.06-1.23)	1.18 (1.09-1.27)	1.15 (1.06-1.24)
Alpha diversity	0.82 (0.61-1.10)	0.97 (0.73-1.29)	0.85 (0.63-1.15)
LRTI Status (ref: no LRTI)			
Indeterminate	1.23 (0.51-2.94)	1.19 (0.50-2.83)	1.21 (0.51-2.87)
CA-LRTI	0.78 (0.38-1.62)	0.76 (0.37-1.58)	0.79 (0.38-1.63)
HA-LRTI	2.14 (0.82-4.24)	1.78 (0.69-4.59)	1.99 (0.76-5.19)
Bacterial genera			
Fusobacterium spp.	2.19 (1.13-4.24)	---	---
Neisseria spp.	---	0.83 (0.45-1.52)	---
Prevotella spp.	---	---	1.63 (0.85-3.11)

	Pseudomonas Model	*Staphylococcus* Model
	**aOR (95% CI)**	**aOR (95% CI)**
Age (years)	1.01 (1.00-1.00)	1.01 (1.00-1.02)
Bacterial abundance	1.17 (1.09-1.26)	1.17 (1.09-1.26)
Alpha diversity	0.90 (0.68-1.20)	0.88 (0.67-1.17)
LRTI Status (ref: no LRTI)		
Indeterminate	1.18 (0.50-2.81)	1.22 (0.51-2.91)
CA-LRTI	0.80 (0.39-1.65)	0.77 (0.37-1.59)
HA-LRTI	1.77 (0.69-4.60)	1.88 (0.73-4.88)
Bacterial genera		
Pseudomonas spp.	1.31 (0.68-2.52)	---
Staphylococcus spp.	---	1.76 (0.94-3.31)

Abbreviations: aOR, adjusted odds ratio; CI, confidence interval; NT rpm, nucleotide reads per million.

Supplemental Table 8. Number of patients in the no lower respiratory tract infection (LRTI) group with detectable antimicrobial resistance gene (ARG) expression and metagenomic detection of a common bacterial respiratory pathogen. Common bacterial respiratory pathogen was defined as the following bacterial species: *Haemophilus influenzae*, *Moraxella catarrhalis*, *Staphylococcus aureus*, *Streptococcus pneumoniae*, *Streptococcus pyogenes*, *Enterobacter spp., Klebsiella spp.*, *Pseudomonas spp.*

	**No detectable ARG**	**Detectable ARG**	**Total**
No respiratory pathogen detected	11	3	14
Detected respiratory pathogen	39	14	53
Total	50	17	67

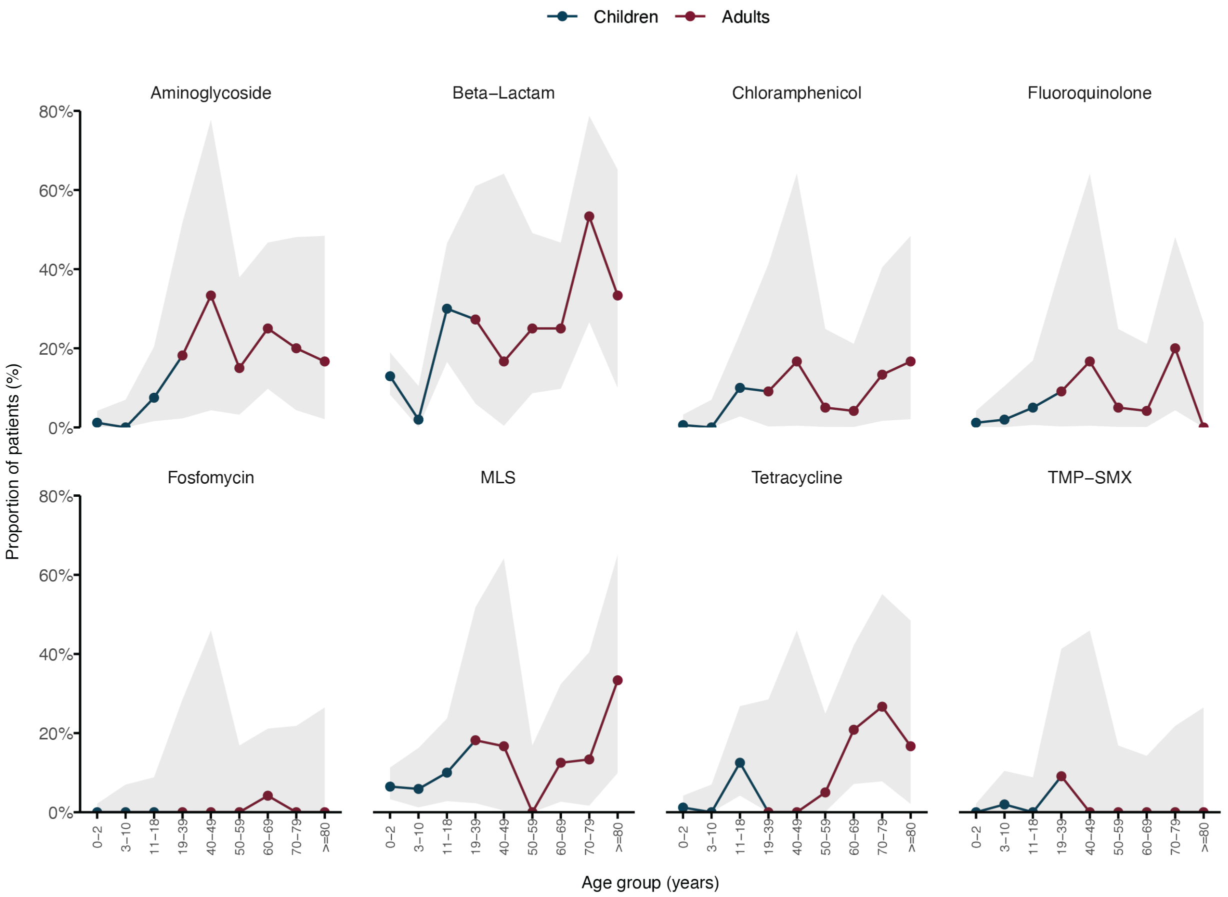

Supplemental Figure 1. Proportion of patients with ARGs by ARG class and age subgroups. 95% confidence intervals (shaded region) were calculated by the Clopper-Pearson exact binomial method. The color indicated children (blue) or adults (red). Abbreviations: MLS, macrolide-lincosamide-streptogramin; TMP-SMX, trimethoprim-sulfamethoxazole.

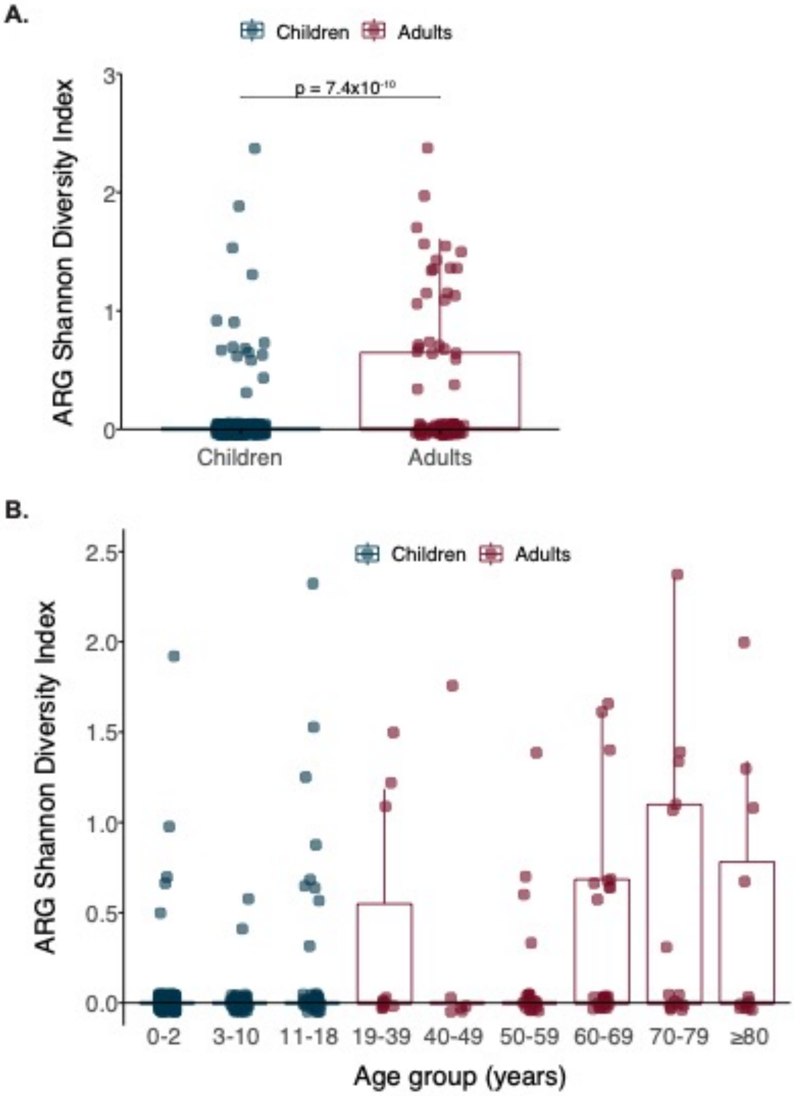

Supplemental Figure 2. Alpha diversity of the resistome by age. Alpha diversity was calculated using the ARG Shannon diversity index for (A) binary age and (B) age subgroups (n = 349 patients). Boxplot elements include a center line (median), box limits (upper and lower quartiles), and whiskers (1.5x interquartile range). Individual data points are shown using overlaid dot plots. In Figure A, the p-value was calculated using two-sided Wilcoxon-rank sum test. The color indicated children (blue) or adults (red).

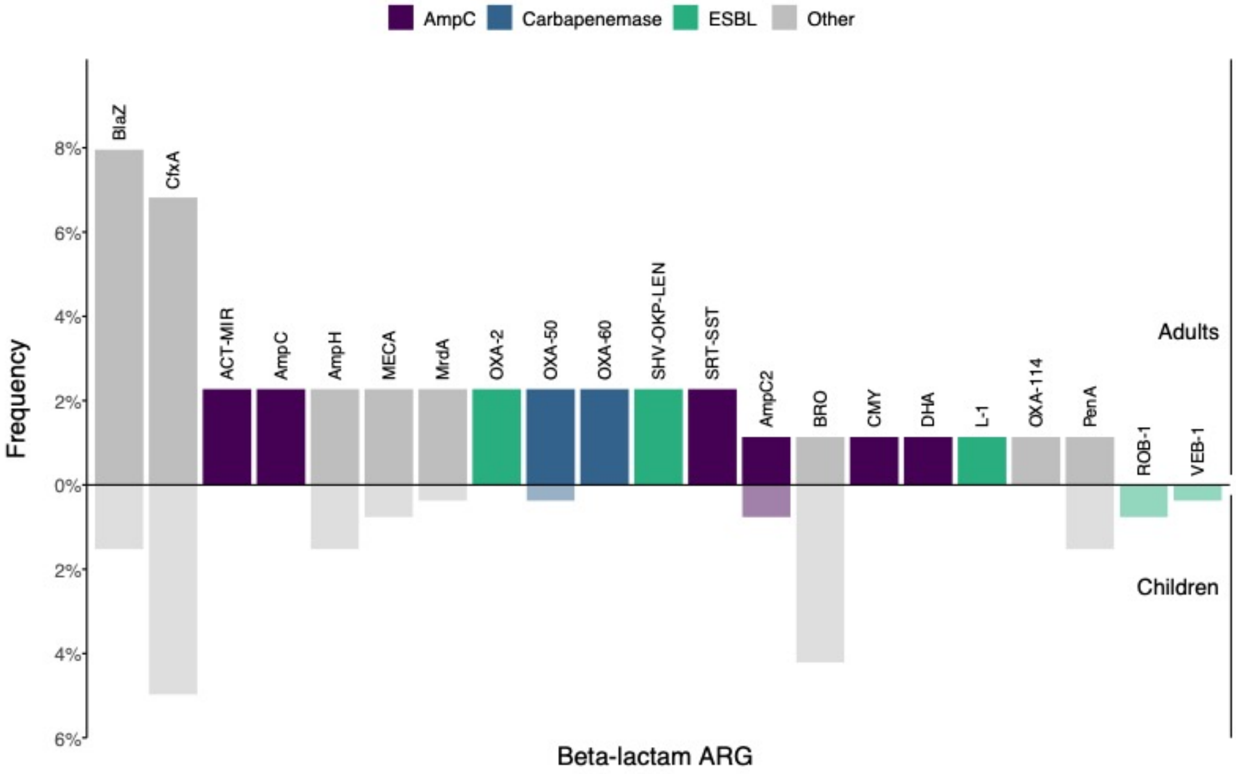

Supplemental Figure 3. Frequency of children (translucent) and adults (solid) with each identified beta-lactam antimicrobial resistance genes (ARGs). Beta-lactam ARGs of particular clinical interest include AmpC beta-lactamases (purple), carbapenemases (blue), and extended spectrum beta-lactamases (ESBL) (green). Beta-lactam ARGs that did not belong to any of these groups were shown in gray.

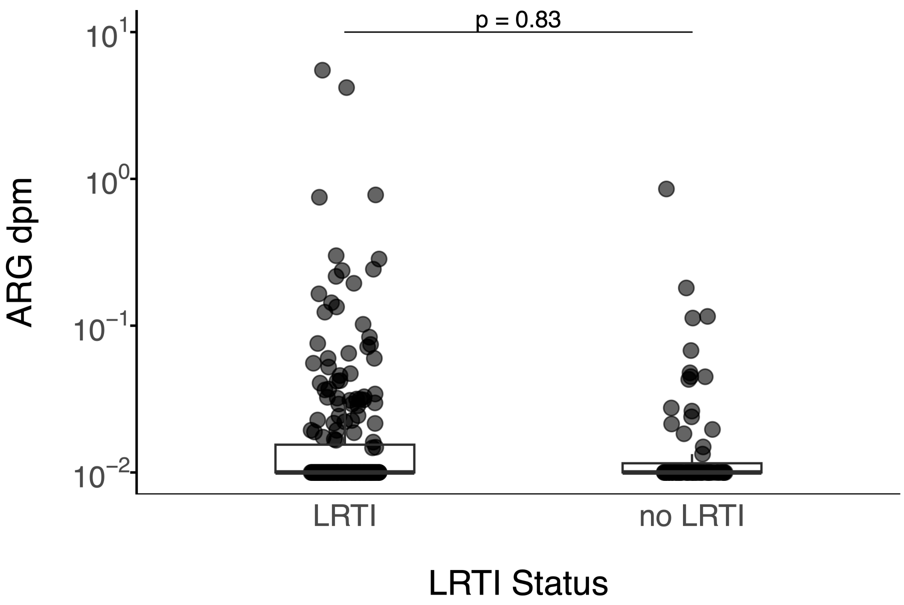

Supplemental Figure 4. Antimicrobial resistance gene (ARG) abundance of patients with lower respiratory tract infection (LRTI) compared with patients no LRTI (n = 349 patients). ARG abundance was normalized by gene length (average ARG sequencing depth per million reads sequenced, dpm). The p-value was calculated using two-sided Wilcoxon-rank sum test. Boxplot elements include a center line (median), box limits (upper and lower quartiles), and whiskers (1.5x interquartile range). Individual data points are shown using overlaid dot plots.

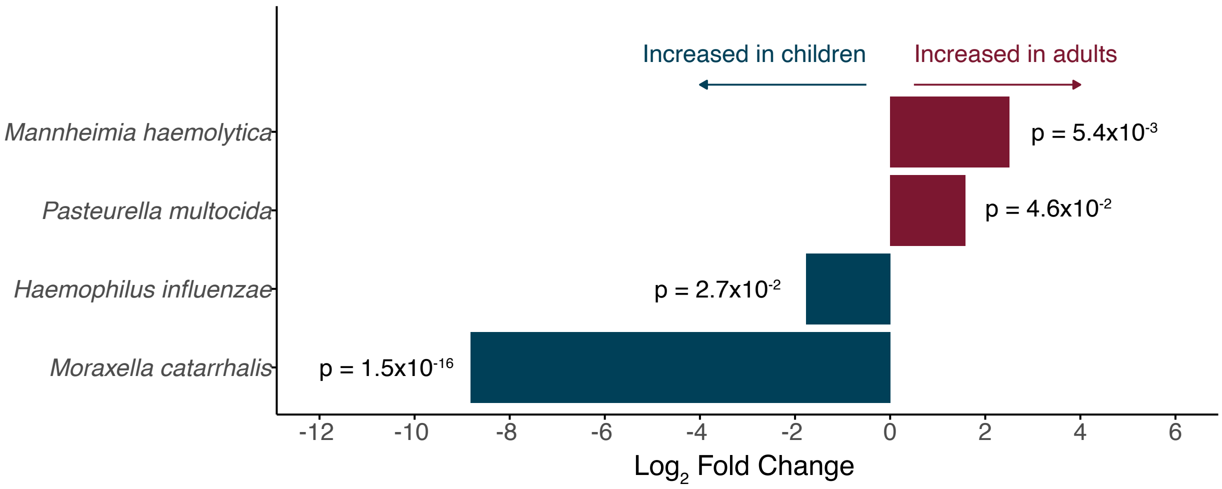

Supplemental Figure 5. Differentially abundance bacterial species in children and adults (n = 349 patients). Statistically significant (Wald's test p-value <0.05) differential abundant bacterial species adjusted for multiple comparisons, by $\log_2$ fold change of bacterial counts, are shown. Bar colors indicate whether the species was more abundant in children (blue) or adults (red).
