# Supplementary material for: The antibiotic resistance reservoir of the lung microbiome expands with age in a population of critically ill patients
Source: Nat Commun. 2024 Jan 2;15:92. doi: 10.1038/s41467-023-44353-1 (PMC10762195; doi:10.1038/s41467-023-44353-1)
Supplement: Supplementary file 3 — Reporting Summary [file 41467_2023_44353_MOESM3_ESM.pdf]

nature portfolio

Corresponding author(s): Charles Langelier

Last updated by author(s): Sep 5, 2023

Reporting Summary

Nature Portfolio wishes to improve the reproducibility of the work that we publish. This form provides structure for consistency and transparency in reporting. For further information on Nature Portfolio policies, see our Editorial Policies and the Editorial Policy Checklist.

Statistics

For all statistical analyses, confirm that the following items are present in the figure legend, table legend, main text, or Methods section.

n/a	Confirmed	
☐	☒	The exact sample size (*n*) for each experimental group/condition, given as a discrete number and unit of measurement
☐	☒	A statement on whether measurements were taken from distinct samples or whether the same sample was measured repeatedly
☐	☒	The statistical test(s) used AND whether they are one- or two-sided<br>*Only common tests should be described solely by name; describe more complex techniques in the Methods section.*
☐	☒	A description of all covariates tested
☐	☒	A description of any assumptions or corrections, such as tests of normality and adjustment for multiple comparisons
☐	☒	A full description of the statistical parameters including central tendency (e.g. means) or other basic estimates (e.g. regression coefficient) AND variation (e.g. standard deviation) or associated estimates of uncertainty (e.g. confidence intervals)
☐	☒	For null hypothesis testing, the test statistic (e.g. *F*, *t*, *r*) with confidence intervals, effect sizes, degrees of freedom and *P* value noted<br>*Give P values as exact values whenever suitable.*
☒	☐	For Bayesian analysis, information on the choice of priors and Markov chain Monte Carlo settings
☒	☐	For hierarchical and complex designs, identification of the appropriate level for tests and full reporting of outcomes
☒	☐	Estimates of effect sizes (e.g. Cohen's *d*, Pearson's *r*), indicating how they were calculated

Our web collection on statistics for biologists contains articles on many of the points above.

Software and code

Policy information about availability of computer code

Data collection	For bulk RNA-seq analyses, following demultiplexing, sequencing reads were pseudo-aligned with kallisto to an index consisting of all transcripts associated with human protein coding genes (ENSEMBL v. 99), cytosolic and mitochondrial ribosomal RNA sequences and the sequences of ERCC RNA standards. Gene-level counts were generated from the transcript-level abundance estimates using the R package tximport, with the scaledTPM method.
Data analysis	Statistical analysis was performed in RStudio, version 2023.06.0+421 using R v4.2.1. Alpha and beta diversity calculations were performed using vegan v2.6.4. Bulk RNA-seq differential expression analysis was performed using DESeq2 package (v1.36.0).<br><br>For microbial analyses, raw sequencing reads underwent quality filtration, removal of human reads and were input into the CZID pipeline version 7.1, which performs reference-based taxonomic alignment at both the nucleotide and amino acid level against sequences in the National Center for Biotechnology Information (NCBI) nucleotide (NT) and non-redundant (NR) databases, followed by assembly of the reads matching each taxon detected.<br><br>Figures were made using the ggplot2 (v3.4.0), epiDisplay (v 3.5.0.2), and patchwork (v 1.1.2) packages for R.

For manuscripts utilizing custom algorithms or software that are central to the research but not yet described in published literature, software must be made available to editors and reviewers. We strongly encourage code deposition in a community repository (e.g. GitHub). See the Nature Portfolio guidelines for submitting code & software for further information.

Data

Policy information about availability of data

All manuscripts must include a data availability statement. This statement should provide the following information, where applicable:

- Accession codes, unique identifiers, or web links for publicly available datasets
- A description of any restrictions on data availability
- For clinical datasets or third party data, please ensure that the statement adheres to our policy

FASTQ files containing non-host reads identified by the CZ-ID pipeline, following subtraction of reads aligning to the human genome, have been deposited in the NCBI Sequence Read Archive (SRA) database under BioProject accessions PRJNA875913 [https://www.ncbi.nlm.nih.gov/bioproject/?term=PRJNA875913] for the pediatric cohort and PRJNA450137 [https://www.ncbi.nlm.nih.gov/bioproject/?term=PRJNA450137] for the adult cohort. The NCBI NT database is available at: ftp://ftp.ncbi.nlm.nih.gov/blast/db/FASTA/. The Antibiotic Resistance Gene-ANNOTation (ARG-ANNOT) database is available at: https://github.com/katholt/srst2/tree/master/data. The processed ARG, bacterial microbiome, and demographic data are available at the Github repository: https://github.com/victoriatchu/agingAMR [DOI: 10.5281/zenodo.10258295]. The source data generated in this study for the figures are provided in the Source Data file with this paper.

Research involving human participants, their data, or biological material

Policy information about studies with human participants or human data. See also policy information about sex, gender (identity/presentation), and sexual orientation and race, ethnicity and racism.

Reporting on sex and gender	Findings apply to both sex (female sex: n=142, male sex: n=206), and data was based on self-reporting and collected from electronic medical records. Sex was included in the multivariable analysis as a covariate, and was not found to be a significant risk factor for presence of antimicrobial resistance genes. Gender was not collected and, as such, gender-based analyses were not performed. However, there are no strong biological reasons for either sex or gender to be a significant risk factor for antimicrobial resistance, and given that sex was not found to be a significant risk factor, we do not expect gender to be a significant risk factor. Disaggregated source data with patient ID, age, and sex is provided.
Reporting on race, ethnicity, or other socially relevant groupings	The race variable and the ethnicity variable were combined and re-categorized as a single variable "race/ethnicity." Categories were "non-Hispanic White" (51%), "Hispanic White" (13%), "Asian" (12%), "Black or African American" (16%), "Other or Multiracial" (6%), and "Missing" (4%). Categories were created based on self-reported race and ethnicity data obtained from the electronic medical record. There was a significant difference in race/ethnicity between the pediatric cohort and the adult cohort. This was addressed in multivariable logistic regression models that included race/ethnicity as a variable. Confounding variables such as enrollment site were accounted for in a sensitivity analysis of just the pediatric cohort (as patient were enrolled from a national pediatric ICU network). We did not use race/ethnicity as a proxy for any other variables (e.g., socioeconomic status).
Population characteristics	Age (261 children, 88 adults) was the main characteristic of interest for this manuscript. Other covariate-relevant population characteristics include lower respiratory tract infection status (67% with a lower respiratory tract infection), geographic region of the patient enrollment site (all adults from the Western U.S. region; 32% of the children were from the Midwest, 47% West, 14% Northeast, and 7% South), and 90% received antibiotics prior to specimen collection. When possible, these variables were included in the multivariable logistic regression analyses.
Recruitment	New ICU admissions were reviewed by clinical research coordinators to determine if they fit the enrollment criteria: mechanically ventilated patients aged 31 days-18 years (pediatric cohort) or 19 years and older (adult cohort) with acute respiratory failure with tracheal aspirate samples obtained within 72 hours of intubation. Written informed consent was obtained from parents or other legal guardians (pediatric patients) and from patients or their surrogates (adult patients), which included permission for collected respiratory specimens and data to be used in future studies. For the adult cohort, the IRB approved of an initial waiver consent for obtaining excess respiratory samples, and informed consent was subsequently obtained for continued study participation according to CHR protocol 10-02701.
Ethics oversight	The pediatric cohort study was approved by a single Institutional Review Board (IRB) at the University of Utah (protocol #00088656). The adult cohort study was approved by the UCSF IRB (protocol #10-02701).

Note that full information on the approval of the study protocol must also be provided in the manuscript.

Field-specific reporting

Please select the one below that is the best fit for your research. If you are not sure, read the appropriate sections before making your selection.

☒ Life sciences ☐ Behavioural & social sciences ☐ Ecological, evolutionary & environmental sciences

For a reference copy of the document with all sections, see nature.com/documents/nr-reporting-summary-flat.pdf

Life sciences study design

All studies must disclose on these points even when the disclosure is negative.

Sample size	Samples were derived from two observational cohort studies (pediatric patients and adult patients who were hospitalized in the ICU for acute respiratory failure, requiring mechanical ventilation). No sample size calculation was preformed as we leveraged this analysis from two robust

	observational cohort studies for analysis. To our knowledge, this is one of the largest cohort studies (n=349) evaluating differences in the antimicrobial resistance genes found in the lung microbiome across all ages (including children and adults). Many microbiome and resistome studies have a much smaller population size (n <50), and our sample size was robust enough for significant findings.
Data exclusions	Water samples were processed in parallel with the patient samples, and we used previously described negative binomial model to exclude microbes likely to be contaminants from the laboratory environment. We also excluded antimicrobial resistance genes with <5% coverage or found in >= 10% of negative water control samples.
Replication	To our knowledge, this is the first lower respiratory RNA-seq dataset of patients across all age ranges (from infant to >90 years old) with acute respiratory failure. As such, no external dataset is available for replication.
Randomization	This is an observational study comparing tracheal aspirate microbial and antimicrobial resistance gene expression in children and adults with acute respiratory failure. As such, patients were note randomized into a given study group.
Blinding	This was an observational cohort study, as such blinding was not applicable. Samples from both cohorts were sequenced on the same sequencing and alignment pipeline, which did not have any information about the subjects.

Reporting for specific materials, systems and methods

We require information from authors about some types of materials, experimental systems and methods used in many studies. Here, indicate whether each material, system or method listed is relevant to your study. If you are not sure if a list item applies to your research, read the appropriate section before selecting a response.

Materials & experimental systems

n/a	Involved in the study
☒	☐ Antibodies
☒	☐ Eukaryotic cell lines
☒	☐ Palaeontology and archaeology
☒	☐ Animals and other organisms
☒	☐ Clinical data
☒	☐ Dual use research of concern
☒	☐ Plants

Methods

n/a	Involved in the study
☒	☐ ChIP-seq
☒	☐ Flow cytometry
☒	☐ MRI-based neuroimaging
